# Development of the active ingredient composition of hand antiseptics in Germany from 2004 to 2022 with special consideration of ethanol as active agent 

**DOI:** 10.3205/dgkh000546

**Published:** 2025-05-02

**Authors:** Philine Grashoff, Nico Tom Mutters, Axel Kramer, Carola Ilschner, Marvin Rausch, Jürgen Gebel

**Affiliations:** 1 Institute of Hygiene and Public Health, University Clinic, Bonn, Germany; 2 Verbund für Angewandte Hygiene, Bonn, Germany; 3 Institute of Hygiene and Environmental Medicine, University Medicine Greifswald, Germany

**Keywords:** active ingredients, hand rubs, development in Germany, increase in ethanol-based hand rubs, decrease in propanol-based hand rubs, waiver of benzoic acid, clorocresol, chlorofene, octenidine dihydrochloride, peracetic acid, polihexanide, triclosan

## Abstract

**Aim:**

The number of active agents used in hand antiseptics (HA) in Germany was analyzed using the disinfectant lists of the Association for Applied Hygiene (VAH) for the years 2004, 2012 and 2022 to evaluate the development regarding the use of unnecessary or critical active agents in alcohol-based hand rubs (ABHR).

**Results:**

While 20 different active agents were used in the HAs (97 listed HAs) in 2004, only 14 were used in 2012 (201 listed HAs) and 15 in 2022 (332 listed HAs). Benzoic acid, clorocesol, chlorophene, octenidine dihydrochloride, peracetic acid, polihexanide and triclosan are no longer used as additives to ABHR. At the same time, the number of active ingredients per product fell.

In the period from 2002 to 2022, there was an increase in ABHR, so that in 2022, only four HAs did not contain alcohol: three were based on PVP iodine and one was based on quaternary ammonium compounds.

While 2-propanol still dominated as the first-named active ingredient in 2004 and 2022, in 2022 mainly ABHR with ethanol as the first-named active ingredient were certified. The percentage share of ethanol in ABHR, measured against all VAH-listed HA and as the main active ingredient, increased by 43.4% between 2004 and 2022. At the same time, there has been a 33.2% decrease in ABHR of 2-propanol as active ingredient.

**Discussion:**

There are probably two reasons for the decrease in the total number of active ingredients used. The addition of antiseptic agents to ABHR does not increase their residual effectiveness. In addition, the antimicrobial antiseptics added to ABHR are less well tolerated than alcohols. Consequently, for ethical reasons it makes sense not to add these antimicrobials to the formulas.

The increase of ethanol-based hand rubs (EBHR) suggests that these are preferred by users. One explanation may be that, unlike ethanol, 1-propanol can have an irritating effect on both healthy and atopic skin.

**Conclusion:**

Ethanol must be retained as an active ingredient for ABHR for the following reasons: ethanol is the only active ingredient that can be used for HA with comprehensive efficacy against non-enveloped viruses; both propanols are less physiological for the human organism than ethanol; ethanol is better tolerated by the skin than 1-propanol; and an adverse effect on the skin microbiome has been ruled out for ethanol. This must be considered when discussing the possible biocide classification of ethanol as CMR, especially because such a classification has absolutely no scientific basis.

## Zusammenfassung

**Zielsetzung:**

Analysiert wurde die Anzahl der in Händedesinfektionsmitteln (HD) eingesetzten Wirkstoffe in Deutschland anhand der Desinfektionsmittel-Listen des Verbunds für Angewandte Hygiene (VAH) der Jahre 2004, 2012 und 2022, um die Entwicklung in Bezug auf den Einsatz nicht erforderlicher bzw. kritischer Wirkstoffe in Alkohol basierten Händedesinfektionsmittel (ABHR) zu bewerten.

**Ergebnisse:**

Während im Jahr 2004 in den HD (97 gelistete HD) 20 unterschiedliche Wirkstoffe eingesetzt wurden, waren es in den Jahren 2012 (201 gelistete HD) nur noch 14 und 2024 (332 gelistete HD) 15 unterschiedliche Wirkstoffe. Nicht mehr eingesetzt werden als Zusatz zu ABHR Benzoesäure, Clorocresol, Clorofen, Octenidindihydrochlorid, Peressigsäure, Polihexanid und Triclosan. Gleichzeitig sank die Anzahl der Wirkstoffe pro Produkt.

Im Zeitraum 2002 bis 2022 zeigte sich eine Zunahme ABHR, so dass 2022 nur noch vier HD keinen Alkohol enthielten, drei auf Basis von PVP-Iod, eins auf Basis von quartären Ammoniumverbindungen.

Während 2-Propanol 2004 und 2022 noch als erstgenannter Wirkstoff dominierte, wurden 2022 hauptsächlich ABHR mit Ethanol als erstgenanntem Wirkstoff zertifiziert. Der prozentuale Anteil von Ethanol in ABHR ist gemessen an allen VAH gelisteten Produkten sowie als Hauptwirkstoff von 2004 bis 2022 um 43,4% gestiegen. Parallel ist ein Rückgang bei ABHR mit der Wirkstoffbasis 2-Propanol um 33,2% zu verzeichnen.

**Diskussion:**

Die Abnahme der Gesamtzahl eingesetzter Wirkstoffe dürfte zwei Gründe haben. Durch Zusatz von antiseptischen Wirkstoffen zu ABHR wird deren remanente Wirksamkeit nicht verstärkt. Zugleich sind die den ABHR zugesetzten antiseptischen Wirkstoffe weniger gut verträglich als Alkohole, so dass der Verzicht ethisch sinnvoll ist.

Der Anstieg Ethanol basierter ABHR spricht dafür, dass diese von den Anwendern bevorzugt werden. Eine Erklärung dürfte sein, dass 1-Propanol im Unterschied zu Ethanol sowohl für gesunde als auch für atopische Haut irritativ wirken kann.

**Schlussfolgerung:**

Da Ethanol als einziger Wirkstoff mit umfassender Wirksamkeit gegen unbehüllte Viren zur HD eingesetzt werden kann, beide Propanole unphysiologischer für den menschlichen Organismus als Ethanol sind, Ethanol besser hautverträglich ist als 1-Propanol und für Ethanol eine Beeinträchtigung des Hautmikrobiom ausgeschlossen wurde, muss Ethanol als Wirkstoff für ABHR erhalten bleiben. Das ist bei der Diskussion der möglichen Biozideinstufung von Ethanol als CMR unbedingt zu berücksichtigen, zumal eine derartige Einstufung der wissenschaftlichen Grundlage entbehrt.

## Introduction

The Commission for Infection Prevention in Healthcare and Nursing Settings (KRINKO) at the Robert Koch Institute (RKI) draws up recommendations for the prevention of healthcare-associated infections (HAIs) in accordance with Section 23 (1) of the Infection Protection Act [1]. These recommendations are continuously developed and published in the Federal Health Gazette of Germany. If the recommendations are followed, compliance with the state of medical science is presumed [1]. 

Hygienic hand antisepsis plays a crucial role in the prevention of both HAIs and communicable infections within the community, as microbial pathogens are primarily transmitted via hands [2]. It is one of the most important measures to protect patients in healthcare and nursing facilities against infection and to protect oneself. Especially since the COVID-19 pandemic, society has become increasingly aware of the importance of hand antisepsis. In its recommendation on hand hygiene in healthcare facilities, the KRINKO refers to the disinfectant list of the Association for Applied Hygiene (VAH) for the selection of disinfectants [3]. In addition, the KRINKO refers to the VAH certification in a statement on requirements for disinfectants and hand antiseptics (HA) for use in all infection-hygiene-sensitive areas [4].

HA have been classified as biocides of product type 1 (PT1) since 2016, but the approval as medicinal products is grandfathered [5]. Due to the human and ecotoxicological requirements for the approval of active agents for biocides, it follows that the composition of the HA available on the market will change. The majority of HA are currently alcohol-based, with ethanol and 2-propanol being the most common active ingredients in alcohol-based hand antiseptics (ABHR). The approval procedure for ethanol under the existing active-agent review program in connection with the Biocidal Products Regulation has not yet been completed (as of January 2025), which is why the transitional rules under Article 89 of Regulation (EU) No. 528/2012 apply [6]. Regardless of how ethanol is classified, it is still possible to place products containing ethanol as an active ingredient on the German market and make them available on the market in Germany without authorization. For these products, registration with the Federal Institute for Occupational Safety and Health has so far been sufficient [7].

Many national and international medical societies and expert committees fear that the current discussion about classifying ethanol as a category 2, if not category 1A or 1B (CMR: carcinogenic, mutagenic, reprotoxic) reproductive toxicant for hand antiseptics that are biocides, will result in banning or at least severly impeding the use of these products for occupational safety reasons. Those bodies demand approval as an active agent for PT1 biocides without this classification [8], [9].

2-propanol was already approved for use in biocidal products for product type 1 in 2014 [10], so that for these products – if they only contain this active agent – an authorization from the European Chemicals Agency (ECHA) must be available [6].

To obtain an overview of how the composition of active agents developed and to better assess the potential effects of a CMR classification of ethanol, the following points were examined on the basis of the VAH lists from the years 2004, 2012 and 2022 in the hygienic HA application area:

- The number of active agents per HA,
- the use of ethanol compared to 2-propanol in the HA.

## Materials and methods

The analysis was based on the printed versions of the VAH disinfectant list and the database for the corresponding certificates from the office of the Disinfectant Commission of the VAH [11]. According to the KRINKO recommendation for hand hygiene [3], products from these lists are used particularly in medical and nursing facilities [4] and are recommended to use in infection-hygiene-sensitive areas [4]. For the area of application of hygienic HA, 97 products were listed in 2004, 201 products in 2012 and 332 products in 2022 [11].

The following criteria were relevant to the analysis:

- Name of the product,
- Active ingredients (listed as substance 1 to substance 4 in the order of nomination in the ingredient list of active substances in the product),
- Percentage of active agents per product, depending on the manufacturer’s specification in percent by weight or volume,
- CAS number of the active agents.

According to Article 3(1)c of the Biocidal Products Regulation (Regulation (EU) No. 528/2012), an active agent is defined as “a substance or micro-organism that has an effect on or against harmful organisms” [5].

For each product, all active ingredients specified by the manufacturer in the certificate (specified excipients were not included) were first listed with the corresponding percentages. These were assigned to active substance groups that are listed as active substance bases in the VAH list [11]: Alcohols, glycol derivatives, guanidines and guanidine derivatives, iodine-releasing compounds, organic acids, peroxide compounds, phenolic derivatives, pyridine derivatives, and quarternary ammonium compounds (QAC). 

If the manufacturers used different names for chemically identical active agents in the applications, such as 2-propanol, isopropanol, isopropyl alcohol or propan-2-ol, these were given the relevant CAS number and harmonized. At the same time, active ingredients with the same name but different concentrations were grouped together (e.g., ethanol 100%, 96%, 93.8%, 80%).

The number of different active agents contained in each product was determined. The active agent listed first in a product always has the largest share in the product composition due to its allocation at the time of application.

Based on this, lists were compiled for the positions of active agent names one to four with the corresponding active agent groups and individual active ingredients of all products. Particular emphasis was placed on the percentage of ethanol and 2-propanol in the products as the first- and second-named active agents. Among other things, this is intended to show whether the use of ethanol as an active agent in alcohol-based hand rubs (ABHR) has changed in relation to 2-propanol.

## Results

It was shown that the total number of active agents used decreased from 2004 to 2012, while it remained constant in the period from 2012 to 2022. While 20 different active agents were contained in the VAH-listed hand rubs in 2004 (97 listed hand rubs), there were only 14 different active agents in 2012 (201 listed hand rubs) and 15 in 2022 (332 listed hand rubs) (Table 1 (Tab. 1)).

**Figure 1:**
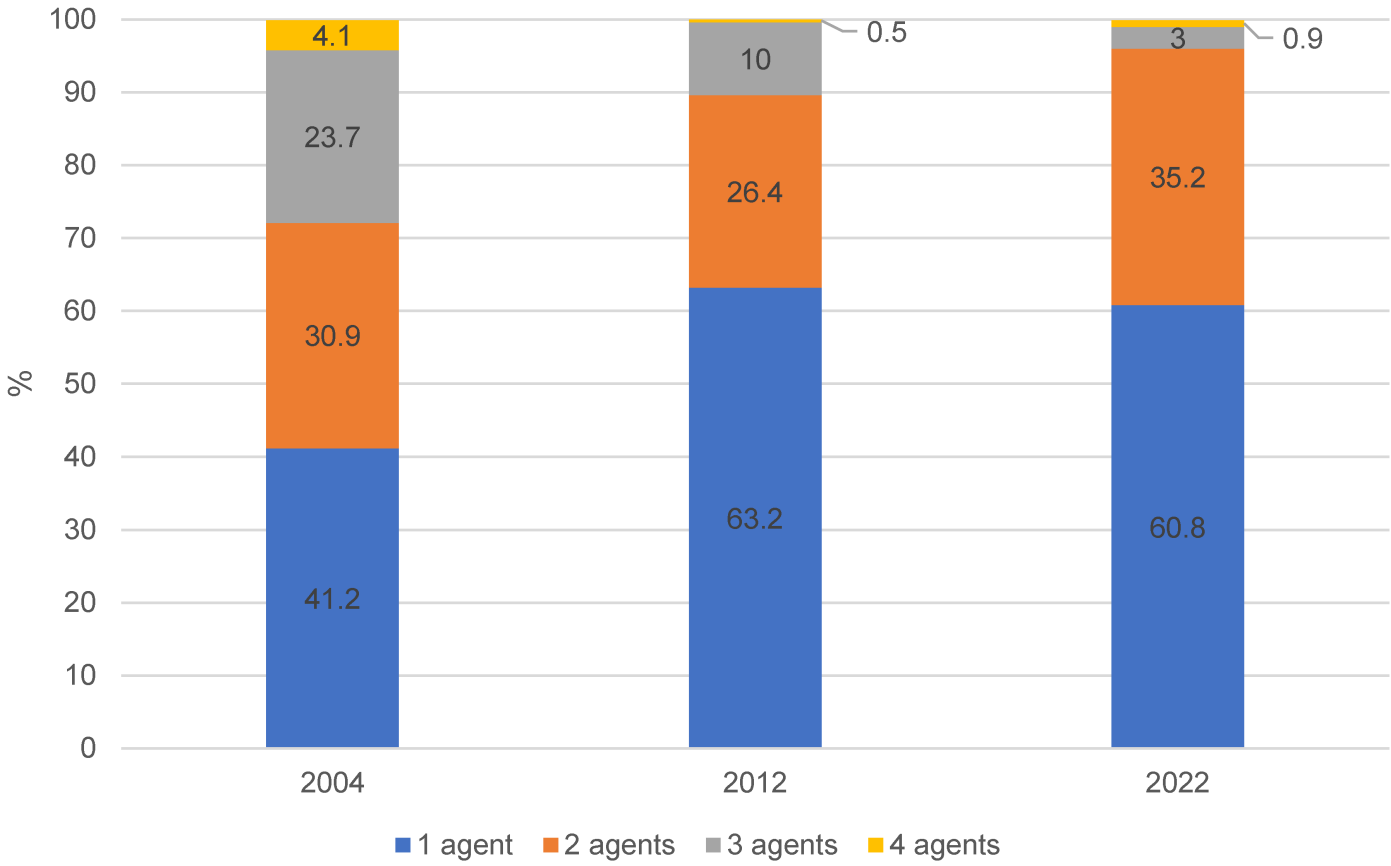
Proportion (%) of hand antiseptics with one, two, three or four active agents in the years 2004, 2012 and 2022

At the same time, the number of active agents per product also decreased between 2004 and 2022. While around 60% of HA still had more than one active agent in 2004, this proportion fell to less than 40% in 2012 and 2022. In 2004, 23.7% of products still had three different active ingredients; in 2012 it was 10% and in 2022 only 3% (Figure 1 (Fig. 1)). 

**Figure 2:**
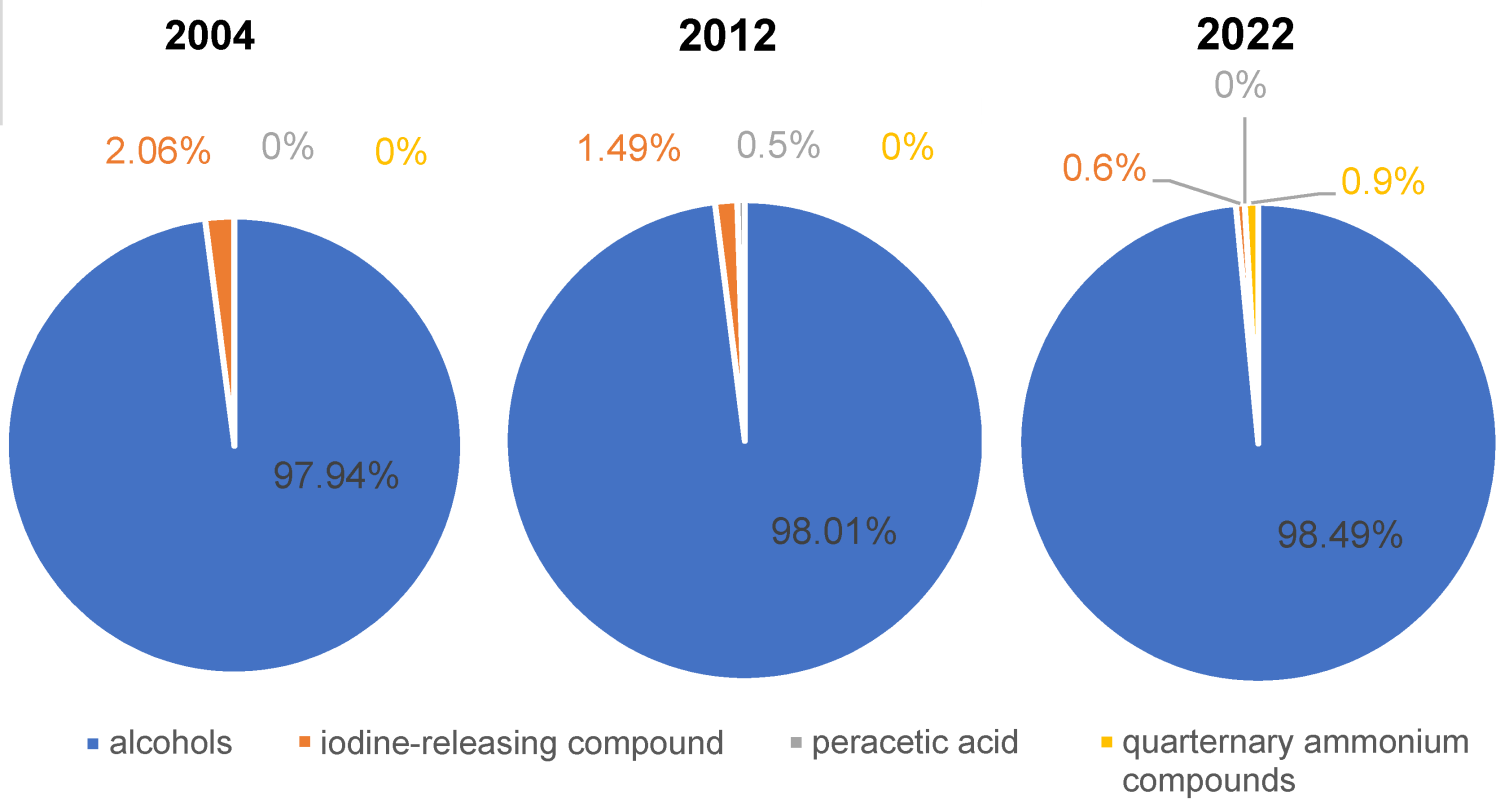
Proportion of first-mentioned active agent (“substance 1”) from the VAH lists of the publication years 2004, 2012 and 2022

If only the first-mentioned active agents (substance 1) of VAH-listed HA are considered, it becomes clear that ABHR are predominant. This trend increases slightly from 2004 to 2022 (Figure 2 (Fig. 2)). 

**Figure 3:**
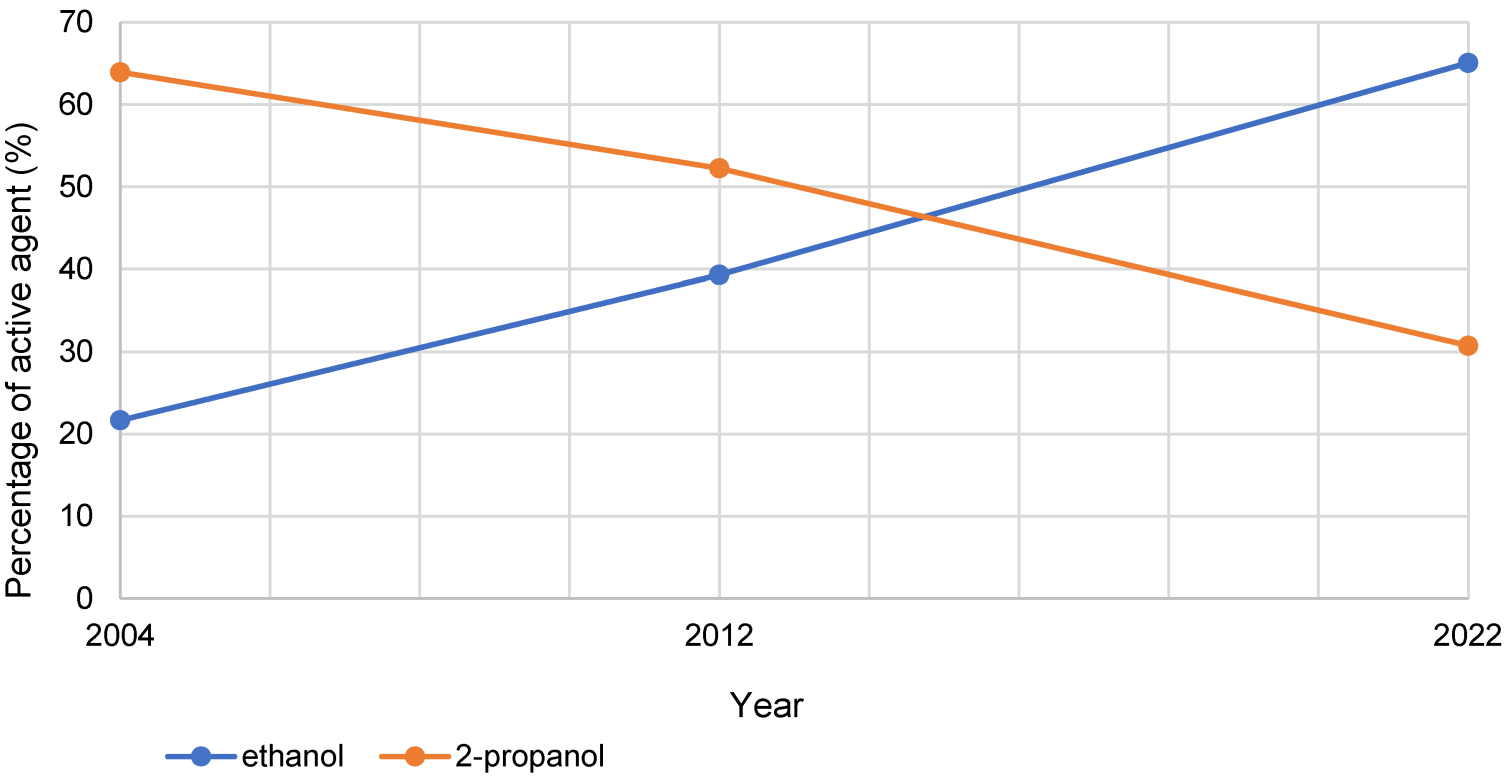
Development of the percentage shares of the active agents ethanol and 2-propanol as “substance 1” over the years 2004, 2012 and 2022

The percentage of ethanol in HA, measured against all VAH-listed HA and as the main active ingredient (“substance 1”), increased by 43.4% between 2004 and 2022. In comparison, there was a 33.2% decrease of 2-propanol as active ingredient. While 2-propanol still dominated as the first-named active ingredient in 2004 and 2022, in 2022 mainly products with ethanol as the first-named active ingredient were certified. This development is shown in Figure 3 (Fig. 3).

## Discussion

There are probably two reasons for the decrease in the total number of active agents used in the period under review from 2004 to 2012:

- Adding antiseptic agents to ABHR does not increase their remanent efficacy [12], i.e., instead of a benefit for the user, it only results in increased costs.
- The active agents used in addition to alcohol are less well tolerated than alcohols, so that ethical considerations demand they be omitted (Table 2 (Tab. 2)).

The authority responsible for processing applications for the approval of biocides in Germany, the Federal Institute for Occupational Safety and Health (BAuA), emphasizes the protection of personnel in healthcare facilities from toxic side effects of biocidal products when granting approval in accordance with the Biocidal Products Ordinance. One of the reasons for the marked reduction of different active ingredients in HA could therefore be the stricter testing of the human and environmental toxicity of the active ingredients. Triclosan, for example, was rejected as an active ingredient for product type 1 in 2016 and can therefore only be found in listed products from 2004.

Consumption of HA is subject to fluctuations. For example, data collected by the ABHR Italian national surveillance system in 2020, 2021 and 2022 showed an overall decline in the consumption of ABHR [13]. While HA use was high at the beginning of the pandemic, adherence to HA practices in Italian healthcare settings gradually declined and had to be rekindled by awareness-raising and training campaigns.

Changes in the use of HA have also been observed in Germany as a result of the COVID-19 pandemic. A sharp increase in demand for HA in both the public and medical sectors initially led to a shortage of products [14]. In order to counteract this, legal suspensions came into force in accordance with Article 55 of the Biocidal Products Regulation, according to which products that do not comply with the Biocidal Products Regulation may also be used for a limited period of time. In March 2020, both the BfArM and the BAuA issued general rulings on the manufacture and use of HA. These allowed the free choice of active-ingredient supplier regardless of the manufacturers on the Article 95 list. As a result, the availability of biocidal products with old active ingredients, including ethanol, increased in the area of hygienic HA [15]. In addition, the BfArM’s general rulings enabled the substitution of non-efficacy-relevant excipients and the free choice of packaging materials and their colors. In order to accelerate the release of preparations, the spore-free specification for medicinal products was suspended [16]. Based on the supply bottleneck for HA, the WHO formulations I and II based on ethanol and 2-propanol with addition of hydrogen peroxide were used for the BAuA’s general rulings so that, in addition to experienced manufacturers, other companies in the pharmaceutical and chemical industries could also produce effective solutions against the enveloped SARS-CoV-2 in order to counteract the shortage [16]. 

The results of the present analysis show an increase in ABHR in the period from 2002 to 2022. In 2022, only four products did not contain alcohol, three of which are based on PVP iodine, and one on QAC. This development is in line with the insufficient effectiveness observed for non-alcohol based hand antiseptics [17] and goes hand in hand with with the observation that ABHR have established themselves worldwide due to their efficacy, spectrum of action and tolerability [18]. The efficacy of ethanol against non-enveloped viruses is comprehensive [8]. The WHO Task Force ABHR and the KRINKO therefore recommend retaining ethanol as a microbicidal active ingredient in ABHR for use in healthcare settings, as it is considered effective and safe for infection prevention and for preventing the development of antimicrobial resistance [8].

An additional cause for the increased consumption of ethanol-based hand rubs (EBHR) suggests that these are preferred by users. One explanation may be that, in contrast to ethanol, 1-propanol can have an irritating effect on both healthy and atopic skin [19].

The Disinfectant Commission of the VAH emphasizes the relevance of ABHR and describes ABHR with the active ingredients ethanol, 1-propanol and 2-propanol as the “gold standard for hand antisepsis” and justifies this with the effectiveness of the products, the skin compatibility, and **the lack of mutagenicity, teratogenicity and carcinogenicity of these active ingredients when used on skin**. In addition to the high efficacy of alcohols, the Disinfectants Commission attaches great importance to the skin compatibility of HA. Against this background, the VAH Disinfectant Commission has published additional requirements as a basis for the certification of non-alcohol-based HA. Products based on QAC or chlorine-based products must either have biocide approval or approval as a medicinal product. This may also have contributed to the increase in ABHR compared to non-alcohol-based hand rubs in the VAH list [20].

As the analysis of the VAH lists shows, ethanol has established itself as the main active ingredient in ABHR. This could be partly due to the fact that in contrast to 1- and 2-propanol, only ethanol is effective against non-enveloped viruses [21].

In 2017, the Canadian Agency for Drugs and Technologies in Health published a review article on the effectiveness of non-alcohol-based hand sanitizers in reducing infection rates and transmission in the healthcare sector. However, only four guidelines and two studies were identified that met the inclusion criteria. The two studies on products with non-alcohol-based active ingredients from France and Finland were unblinded and non-randomized. One study was on a chlorhexidine-based product and the other on a polihexanide-based product. No statements were made on the effectiveness of these products with regard to infection rates. In contrast, there are four evidence-based guidelines with recommendations for the selection of hand antiseptic in the healthcare sector. The National Institute for Clinical Excellence (NICE) advocates the use of ABHR as a critical factor in infection prevention, as does the WHO guideline on hand hygiene. The Public Health Agency of Canada also recommends ABHR as the preferred method for hand hygiene and, like the authors of Public Health Ontario in “Best Practices for Hand Hygiene in All Health are Settings”, explicitly advocates against the use of non-alcohol-based hand sanitizers in all healthcare settings [22]. 

With regard to toxicological concerns about ethanol as an active ingredient, Kramer et al. [8] refer in their 2022 statement to the fact that the consumption of non-alcoholic beer, flavored water and apple juice can lead to similar or even higher ethanol concentrations in the blood than after hand antisepsis with EBHR. This underlines the safety of topical application of EBHRs.

In addition, it should be borne in mind that ethanol is currently still an existing active ingredient, meaning that it is also protected in Germany as an active ingredient in medicinal products without biocide registration or authorization. These aspects could also have led to an overrepresentation of ethanol.

Regulation (EU) No 528/2012 [6] provides that active agents may be approved in exceptional cases for a maximum period of five years, provided that they meet at least one of the conditions set out in Article 5(2). These conditions include a negligible risk to humans, animals or the environment from exposure to the active agent in a biocidal product (Article 5(2)(a) of Regulation (EU) No 528/2012) [6]. 

## Conclusions

The analysis of the development of the use of active agents in VAH-certified products for hygienic hand antisepsis revealed a significant reduction in the number of active agents used. It should be noted that no new active agents have been added and that the first active ingredient mentioned is primarily alcohol with an upward trend from 2004 to 2022. This was accompanied by a simultaneous decline in HA with the active agent 2-propanol.

The possible classification of ethanol as a CMR substance could lead to fewer manufacturers of EBHR registering their product for approval as a biocide. This would result in the loss of HA with efficacy against non-enveloped viruses. There are major differences between the three alcohols in terms of metabolically mediated physiological blood levels. The increase in blood concentrations above baseline after use of EBHR is approximately 157-fold, but after use of 1- and 2-propanol based hand rubs it increases >1,800- and >10,000-fold, respectively [23]. This means that both propanols are less physiological than ethanol and it remains to be seen whether, unlike ethanol [24], they may damage the skin microbiome. 

The availability of raw materials, their sustainability and the development of tolerances to biocidally active substances will presumably also have an influence on which active agents will assert themselves or become established for hygienic hand antisepsis in Germany and other countries in the future.

The assessment for the use of ethanol as a biocidal is currently being carried out by Greece as the rapporteur Member State and the harmonized classification as CMR is being discussed [25]. Consequences for such CMR substances (carcinogenic, mutagenic, reprotoxic) could be, for example, restrictions on use. Accordingly, these substances may not be placed on the market or used if they exceed certain concentration limits [26]. To be exact, a CMR classification for the approval of active agents would mean that ethanol could only be approved as a biocidal for a period of five years in accordance with Article 4 of the Biocidal Products Regulation and supplied to healthcare facilities [6]. For the approval of biocidal products, a CMR classification would mean that these biocidal products would not be approved for use by the general public if the concentration exceeded 0.1%. Affected manufacturers would presumably replace the active ingredient ethanol with other approved active ingredients such as 1- or 2-propanol, which would lead to considerable challenges due to the specificity of ethanol towards non-enveloped viruses. In addition, it remains questionable whether the necessary hygiene standards in the healthcare sector can be maintained with the classification of ethanol as CMR, as ethanol is more effective than the propanols mentioned in combating non-enveloped viruses [27] and more skin tolerable than 1-propanol [19]. 

In the event of a positive assessment, i.e., no CRM classification by the rapporteur Member State, the use of EBHR could increase further as a result. 

## Notes

### Competing interests

The authors declare that they have no competing interests.

### Authors’ ORCID

- Grashoff P: https://orcid.org/0009-0005-5358-4710
- Mutters NT: https://orcid.org/0000-0002-0156-9595
- Kramer A: https://orcid.org/0000-0003-4193-2149
- Ilschner C: https://orcid.org/0009-0006-4083-7405
- Rausch M: https://orcid.org/0000-0003-1562-4337
- Gebel J: https://orcid.org/0000-0001-9328-3174

### Funding

No funding.

## Figures and Tables

**Table 1:**
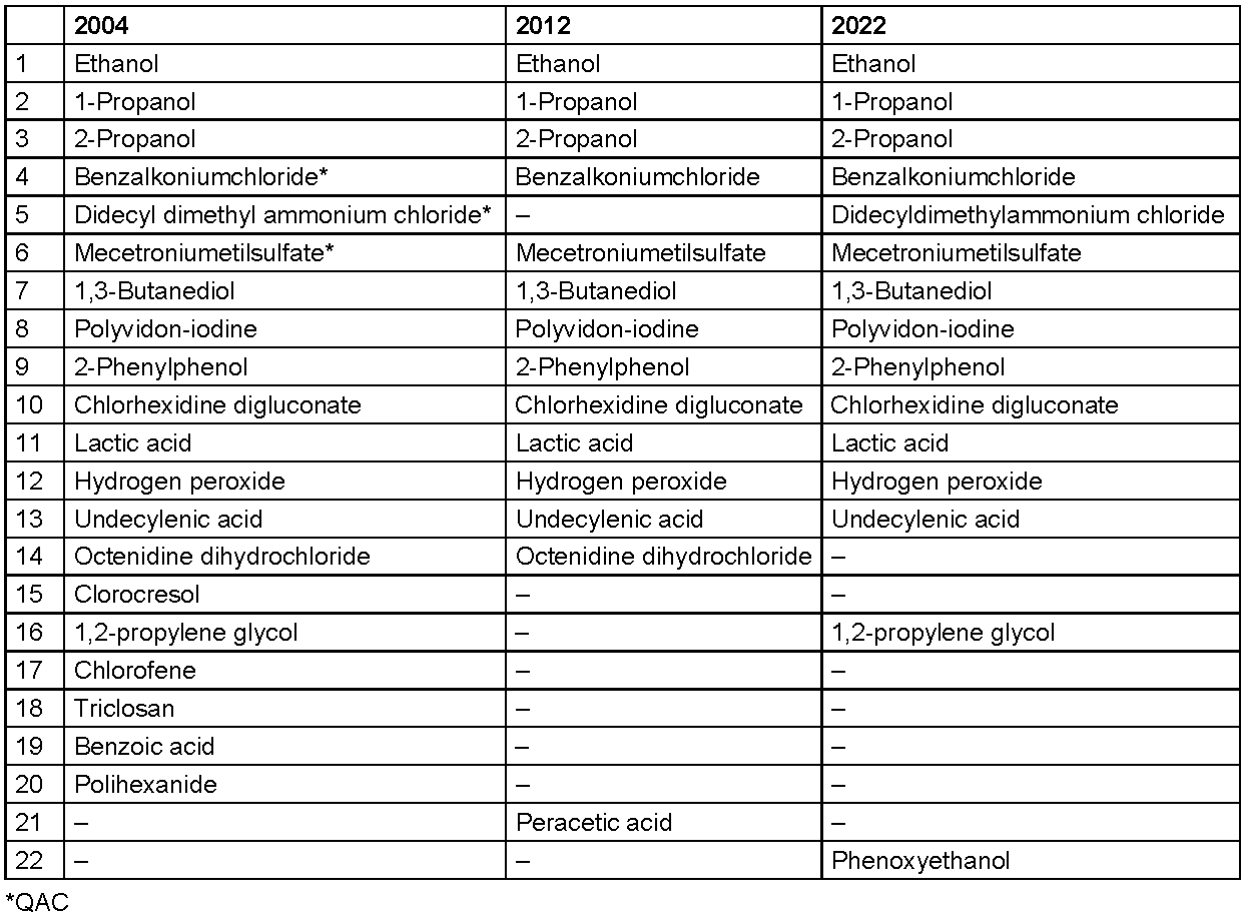
Number of active agents contained in alcohol-based hand rubs in the lists of the Association for Applied Hygiene

**Table 2:**
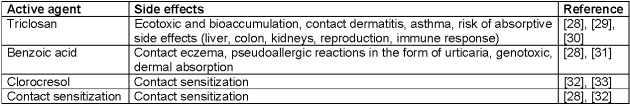
Risk assessment of active agents no longer used in alcohol-based hand rubs since 2012
